# Inflammatory Myofibroblastic Tumor of the Lung: An Extremely Rare Condition in Adults

**DOI:** 10.7759/cureus.6432

**Published:** 2019-12-20

**Authors:** Ammar Al-Obaidi, Charles Buess, Job Mogire, Pavan S Reddy

**Affiliations:** 1 Internal Medicine, Kansas University School of Medicine, Wichita, USA

**Keywords:** inflammatory myofibroblastic tumor, inflammatory pseudotumor, pseudotumor, lung tumors

## Abstract

Inflammatory myofibroblastic tumors (IMTs) of the lung were first reported in 1939. The most common site of predilection is the lungs of the pediatric population. They are extremely rare in adults, constituting less than 1% of adult lung tumors. They are mesenchymal neoplasms that may arise in the soft tissues of almost every organ. IMTs often arise from excessive inflammatory response, and as the name implies, they are composed of myofibroblastic spindle cells accompanied by an inflammatory infiltrate of plasma cells, lymphocytes, and eosinophils.

## Introduction

Inflammatory myofibroblastic tumors (IMTs) are mesenchymal neoplasms that may arise in the soft tissues of almost every organ [1]. IMTs often arise from excessive inflammatory response, and as the name implies, they are composed of myofibroblastic spindle cells accompanied by an inflammatory infiltrate of plasma cells, lymphocytes, and eosinophils.

IMTs of the lung were first reported in 1939 [2]. The most common site of predilection is the lungs of the pediatric population. They are extremely rare in adults, constituting less than 1% of adult lung tumors. No gender preference has been reported in terms of developing these tumors and no geographic or ethnic predispositions [3].

IMTs may be benign, invade surrounding structures, undergo malignant transformation, recur, or even metastasize. This makes the clinical presentation to be of a wide spectrum. Patients may be asymptomatic or present with cough, hemoptysis, dyspnea, pleuritic pain, constitutional symptoms, or pneumonia [1].

Due to such disparities in tumorous process and nonspecific presentation, the clinical and radiologic diagnosis of lung IMTs is very difficult and this confounds treatment due to the highly unpredictable biologic behavior of these tumors [2].

## Case presentation

**Figure 1:**
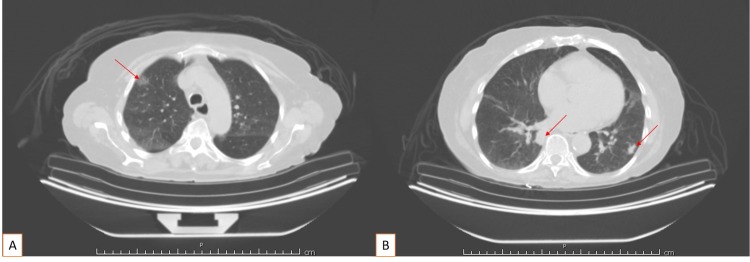
CT scan of the chest with contrast 1.3 cm opacity in the right upper lobe (A); 2.3 cm nodule in the right lower lobe and 1.4 cm nodule in the left lower lobe (B) Several additional pulmonary nodules are identified within the left lower lobe and lingula; no significant pleural effusion; no pneumothorax; scattered vascular calcifications

An 84-year-old Caucasian female with a past medical history of osteoarthritis, peripheral neuropathy, and hypothyroidism presented for persistent non-productive cough. Her cough was described as dry and had been present approximately for two months. She had no smoking history. Lab values were significant for a WBC count of 2.9 x 10^9^/L and hemoglobin (Hgb) of 9.6 gm/dl. All other lab values were unremarkable. Workup was initiated and chest X-ray showed patchy bibasilar infiltrates, right greater than left. CT scan of the chest was abnormal, showing 1.3 cm opacity in the right upper lobe, a 2.3 cm nodule in the right lower lobe, a 1.4 cm nodule in the left lower lobe, and several additional nodules within the left lower lobe and lingula (Figure 1).

**Figure 2:**
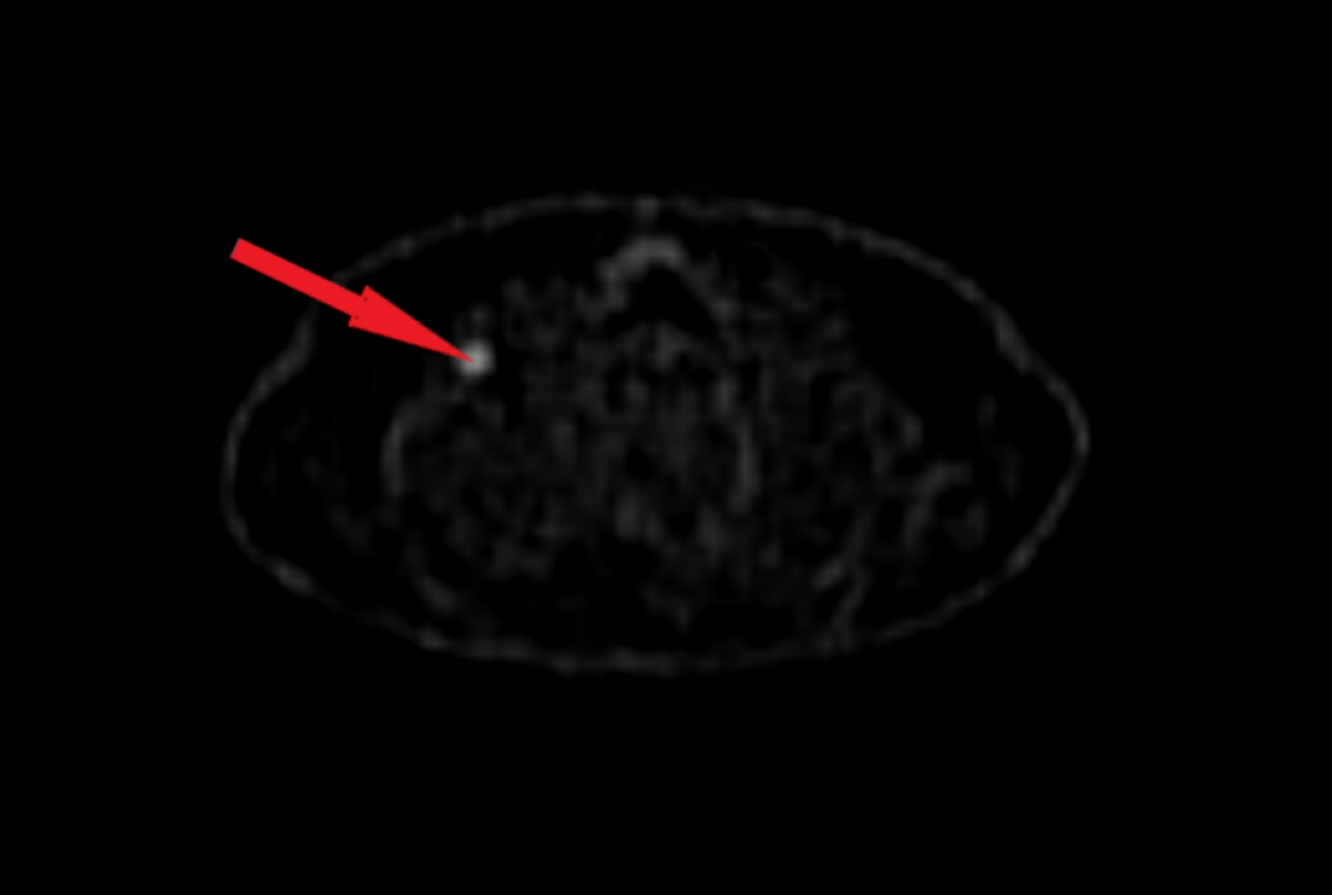
CT/PET scan A hypermetabolic nodule in the right lower lobe CT/PET: positron emission tomography-computed tomography

Subsequent positron emission tomography-computed tomography (CT/PET) showed two hypermetabolic right lower lobe nodules with associated bilateral suprahilar hypermetabolic lymph nodes with associated bilateral hilar metastatic disease (Figure 2). Multiple left-sided nodules were noted, none with suspicious hypermetabolic activity.

**Figure 3:**
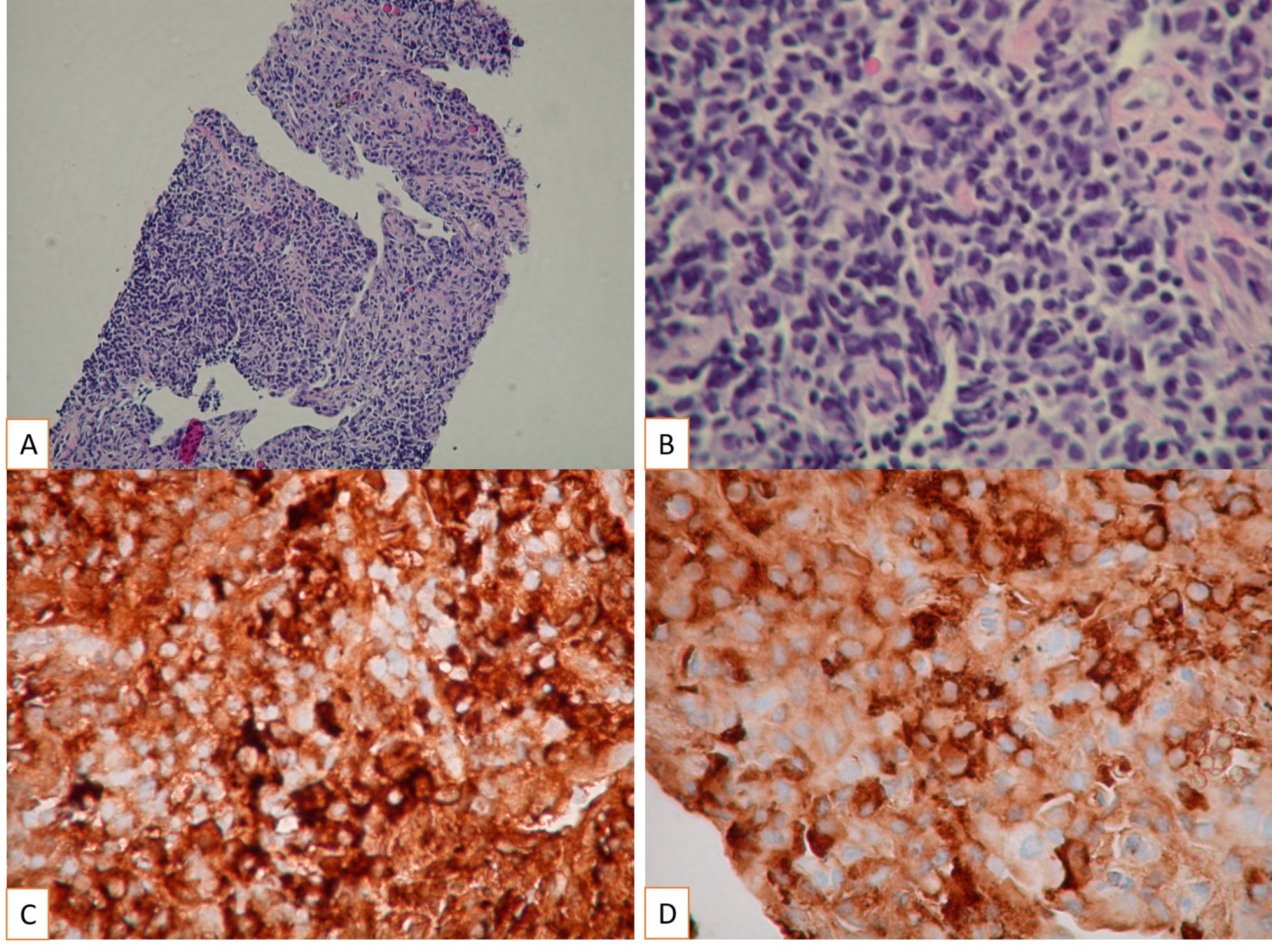
The pathology specimen demonstrates the characteristic features, including spindle-shaped cells with a high nucleocytoplasmic ratio, pleomorphic hyperchromatic nuclei, brisk mitosis without atypia and intense inflammatory cell infiltrate, especially plasma cells Morphology of IMT demonstrated (H&E stain A: 100X magnification and B: 400X magnification), (Immunohistochemical staining C: Kappa 400X magnification and D: Lambda 400X magnification) IMT: inflammatory myofibroblastic tumors; H&E: hematoxylin and eosin

Biopsy of the lesion from the left lower lobe of the lung was consistent with features of inflammatory pseudotumor with plasma cell variant (Figure 3).

The decision was made to proceed with surveillance alone. Repeat CT six months later showed that several subsolid densities in the lung bilaterally had not appreciably changed. A more solid nodule at the left base was also unchanged. The plan is for continued surveillance with a return to the clinic in one year.

## Discussion

Inflammatory myofibroblastic tumors are mesenchymal neoplasms that may develop in nearly every organ. Though IMTs account for 20%-50% of all pediatric primary lung tumors, they represent less than 1% of lung tumors in adults [2,4]. Because of uncertainty regarding the true biologic origin of these tumors, other synonymous names used to refer to IMTs include plasma cell granuloma, xanthogranuloma, inflammatory myofibroblastic tumor, inflammatory pseudotumor, fibroxanthoma, and fibrous histiocytoma [2,5].

Despite the multiplicity of nomenclature, the pathologic hallmark of IMTs is a varying degree of inflammatory cells (lymphocytes, plasma cells, histiocytes, and occasional eosinophils) on a background of bland sheets of proliferating spindle cells with limited cytologic atypia and minimal mitotic activity. Nevertheless, it can be difficult to differentiate IMTs from infections such as atypical mycobacteria, syphilis, and other granulocytic diseases. Some researchers have attempted to subdivide IMTs into subgroups such as organizing pneumonia, fibrous histiocytoma, and lymphoplasmacytic type based on the predominant histocytopathology [2].

A further diagnostic challenge is the fact that IMTs can mimic nonspecific inflammatory reactions, such as pseudolymphoma, immunoglobulin G (IgG)-4 related disease, and cryptogenic organizing pneumonia [2,6]. Because of the diverse nature of the tissue of origin, most IMTs immunostain positive for desmin, caldesmon, and anaplastic lymphoma kinase-1 (ALK-1) and often for smooth muscle actin (SMA), keratin cocktail, and S100. Immunohistochemical staining helps distinguish IMT from tumors of similar histopathology and expression of ALK-1 being highly specific for IMTs.

Most patients (70%) diagnosed with IMTs have asymptomatic disease that is found incidentally on imaging, preceded by or concurrent with a respiratory infection in only about 30% of patients [2]. Clinical symptoms of IMTs are oftentimes nonspecific; they include cough, atypical chest pain, hemoptysis, shortness of breath, fever, fatigue, and rarely anorexia, night sweats, and weight loss [4]. Laboratory findings are equally nonspecific and may include anemia, thrombocytosis, and an elevated sedimentation rate.

Whereas localized disease diagnosed early is amenable to complete resection with a benign prognosis, locally invasive, recurrent, and metastatic IMTs present challenges of malignant disease. Tumors amenable to complete surgical resection and small tumors carry a favorable prognosis and improved survival [7]. Following complete resection, only 2% of patients present with recurrence as compared to 60% recurrence after incomplete resection [2]. Tumors that are multifocal or located proximal to vital organs and vasculature or locally invasive tumors might be unresectable and may be deemed suitable for partial resection, medical therapies (including glucocorticoids and chemotherapy), or radiotherapy. Evidence for the effectiveness of treatments other than resection is still limited.

## Conclusions

Because of the highly variable clinical presentation of IMTs, it remains a matter of debate as to whether IMTs represent an inflammatory response to malignancy or whether IMTs are a primary inflammatory process. The lack of decisive criteria for the diagnosis and guidelines for the management, in addition to the low prevalence of IMTs in the adult population, implies that most cancer centers lack experience and/or guidance for diagnosing and treating these neoplasms.
